# Relationship of circulating cell-free DNA levels to cell-free fetal DNA levels, clinical characteristics and laboratory parameters in preeclampsia

**DOI:** 10.1186/1471-2350-10-120

**Published:** 2009-11-21

**Authors:** Levente Lazar, János Rigó, Bálint Nagy, Krisztián Balogh, Veronika Makó, László Cervenak, Miklós Mézes, Zoltán Prohászka, Attila Molvarec

**Affiliations:** 1 1st Department of Obstetrics and Gynecology, Semmelweis University, Budapest, Hungary; 2 Research Group of Animal Breeding and Hygiene, Faculty of Animal Science, University of Kaposvár, Kaposvár, Hungary; 3 3rd Department of Internal Medicine and Szentágothai Knowledge Center, Semmelweis University, Budapest, Hungary; 4 Research Group of Inflammation Biology and Immunogenomics, Hungarian Academy of Sciences, Budapest, Hungary; 5 Department of Nutrition, Faculty of Agricultural and Environmental Sciences, Szent István University, Gödöllő, Hungary

## Abstract

**Background:**

The aim of our study was to examine whether increased circulating total cell-free DNA levels are related to the clinical characteristics and standard laboratory parameters of preeclamptic patients, to markers of inflammation, endothelial activation or injury, oxidative stress and to cell-free fetal DNA levels.

**Methods:**

Circulating total cell-free DNA was measured by real-time quantitative PCR in plasma samples obtained from 67 preeclamptic and 70 normotensive pregnant women. Standard laboratory parameters, C-reactive protein, plasma von Willebrand factor antigen, plasma fibronectin, plasma malondialdehyde and cell-free fetal DNA levels were also determined.

**Results and Conclusion:**

Circulating total cell-free and fetal deoxyribonucleic acid levels were significantly elevated in pregnancies complicated by preeclampsia (median: 11.395 vs. 32.460 and 0.001 vs. 0.086 pg/μl; P < .001). The quantity of plasma total cell-free DNA did not correlate with most of the laboratory parameters, except for serum aspartate aminotransferase and alanine aminotransferase activities (correlation coefficient: 0.31; P = 0.012 and 0.46; P < .001). There was no correlation with clinical characteristics, including body mass index. The releases of both free fetal and total cell-free deoxyribonucleic acid were found to be affected in preeclampsia. Hepatocellular necrosis seems to be responsible - at least partly - for increased circulating total DNA levels in preeclampsia, as suggested by the significant correlation with liver enzyme activities.

## Background

Preeclampsia is one of the leading causes of maternal and perinatal morbidity and mortality in the developed world [1,2]. It is characterized by hypertension and proteinuria developing after midgestation in previously normotensive pregnant women. Although the exact etiology of preeclampsia remains elusive [2], there appears to be a defect in trophoblast invasion with diminished infiltration and modification of the spiral arteries leading to impaired placentation and subsequent uteroplacental insufficiency [3]. The ischemic and oxidatively stressed placenta releases proinflammatory (Th1) cytokines, lipid peroxidation products and trophoblast debris (syntitiotrophoblast microfragments, cytokeratin, soluble DNA and RNA of fetal origin and even trophoblast cells) into the maternal circulation, which in turn trigger an excessive maternal systemic inflammatory response [4-6]. The systemic inflammatory response with systemic oxidative stress and generalized endothelial dysfunction appears to be the cause of the maternal syndrome of preeclampsia [6]. Several maternal and familiar factors such as maternal age and body mass index, chronic hypertension or renal diseases, and hypertension in family history have been described as risk factors of preeclampsia. In the last ten years, several studies have reported the increased level of fetal cells, cell-free maternal and fetal DNA in the maternal circulation [7]. While the latter seems to reflect placental injury, the reason for increased cell-free maternal DNA levels in preeclampsia is currently unknown [8,9]. The aim of our study was to measure and compare the concentration of cell-free (cf) and cell-free fetal (cff) DNA levels in the plasma of preeclamptic and normotensive pregnant women, and to determine whether increased circulating cf DNA levels are related to clinical characteristics and standard laboratory parameters of preeclamptic patients, to markers of inflammation (C-reactive protein), endothelial activation (von Willebrand factor antigen) or endothelial injury (fibronectin), oxidative stress (malondialdehyde) and to cell-free fetal DNA levels.

## Methods

### Patients

This study was performed in a retrospective manner. Sixty-seven plasma samples were collected from women with preeclampsia during pregnancy; these samples were stored frozen. Preeclampsia was determined by a blood pressure of ≥ 140/90 mmHg and an associated proteinuria of ≥ 300 mg/24 h after 20 weeks' gestation. Seventy samples were taken from normotensive women with normal pregnancies. All women were pregnant with a single fetus; 36 of those with preeclampsia had a single male fetus, as did 25 of the control cohort. Pregnant women with eclampsia or HELLP syndrome (hemolysis, elevated liver enzymes, and low platelet count) were not enrolled in this study. The study protocol was approved by the Regional Institutional Committee of Medical Ethics at the Semmelweis University, and written informed consent was obtained from each patient. The study was conducted in accordance with the Declaration of Helsinki.

### DNA extraction and PCR analysis

Blood was collected into sterile EDTA tubes, and plasma was rapidly separated by centrifugation at 3000 *g *and stored frozen at -80°C until the analyses were performed. DNA was extracted from 400 μL plasma with the High Pure PCR Template Preparation Kit (Roche Diagnostics, Mannheim, Germany) according to the manufacturer's protocol. Strict anticontamination procedures were used throughout. These included the use of aerosol-resistant tips throughout all the experimental procedures and the addition of multiple negative control samples in each analysis. The DNA was eluted in 50 μL of elution buffer solution, of which 2 μL was used as a template for the PCR reaction.

### SYBR Green PCR analysis

For the SYBR Green real-time PCR analysis, we used a LightCycler 1.0 System (Roche Diagnostics, Mannheim, Germany). Circulating male fetal DNA was detected with the following primers for the *SRY *gene located on the Y chromosome: forward, SRY F, 5'-ggc aac gtc cag gat aga gtg a-3', reverse 5'-tgc tga tct ctg agt ttc gca tt-3'. To determine the total amount of circulating DNA present in the maternal plasma samples, we used a SYBR Green assay and primers for the globin gene, which is present in all genomes. In this analysis, the following primers were used: forward 5'-aca caa ctg tgt tca cta gc-3', reverse 5'-caa ctt cat cca cgt tca cc-3'. The PCR analysis was carried out in 10-μL reaction volumes containing 1 μl DNA, 2,5 pmol/L of each amplification primer, 2 μl of DNA Master SYBR Green I mix (LightCycler FastStart DNA Master SYBR Green I kit: Taq polymerase, dNTP, MgCl_2_), and 6 μl of nuclease free water.

The analysis was designed in such a manner that identical thermal profiles could be used for both the *SRY *and globin SYBR Green assays, because this allowed us to analyze both of these markers on the same plate in the same analytic run. The PCR was carried out in 40 cycles under the following conditions: initial denaturation 8 min on 95°C, 5 sec denaturation on 95°C, 10 sec annealing on 60°C, 15 sec extension on 72°C, cooling to 4°C till capillar removement. To determine the number of copies of circulating DNA present in the plasma sample, a standard dilution curve with a known concentration of male genomic DNA was used. All samples were analyzed in duplicate and scored in a blinded manner.

### Laboratory parameters

Standard laboratory parameters (clinical chemistry) and C-reactive protein (CRP) levels were determined by an autoanalyzer (Cobas Integra 800, Roche, Mannheim, Germany) using the manufacturer's kits. Plasma von Willebrand factor antigen (VWF:Ag) levels were quantified by ELISA (Dakopatts, Glostrup, Denmark), while plasma fibronectin concentration by nephelometry (Dade Behring, Marburg, Germany), according to the manufacturer's instructions. Plasma malondialdehyde levels were measured by the thiobarbituric acid-based colorimetric assay [10].

### Data analysis

The normality of continuous variables was assessed using the Shapiro-Wilk's *W*-test. As the continuous variables were not normally distributed, nonparametric statistical methods were used. To compare continuous variables between the two groups, the Mann-Whitney *U*-test was applied, while to compare categorical variables between the groups, the Fisher exact and Pearson χ^2 ^tests were performed. As plasma total and fetal DNA levels showed skewed distributions, we performed analyses of covariance (ANCOVA) with logarithmically transformed data. The Spearman rank order correlation was used to calculate correlation coefficients. Statistical analyses were carried out using the following software: STATISTICA (version 7.1; StatSoft, Inc., Tulsa, Oklahoma, USA) and Statistical Package for the Social Sciences (version 15.0 for Windows; SPSS, Inc., Chicago, Illinois, USA). For all statistical analyses, p < 0.05 was considered statistically significant.

## Results

In this study, performed retrospectively and in a blinded manner, 67 plasma samples were obtained from pregnant women with preeclampsia. Thirty-six of them carried male fetuses (gestational age at blood draw, median (range): 37.5 (30-41)). Seventy plasma samples were obtained as control from normotensive pregnant women, 25 of whom carried male fetuses (gestational age at blood draw, median (range): 36 (29-39)).

**Figure 1:**
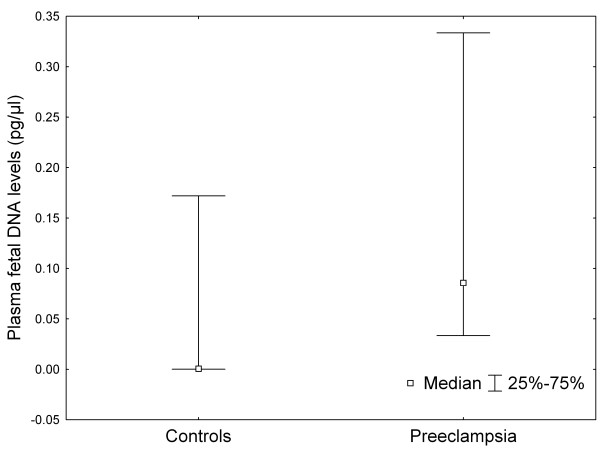
**Plasma fetal deoxyribonucleic acid (DNA) levels in normotensive, healthy pregnant women and preeclamptic patients**. Middle point: median, whisker: 25-75 percentile.

**Figure 2:**
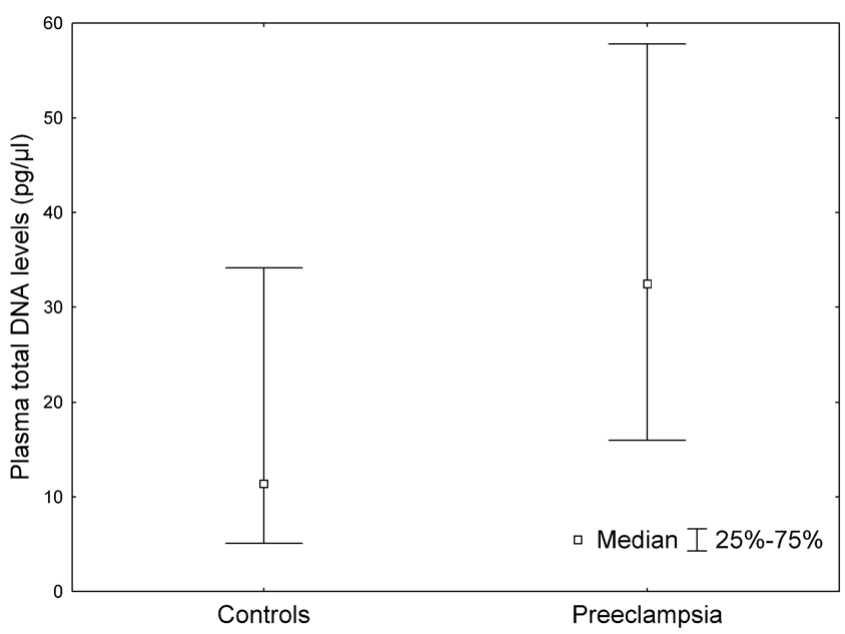
**Plasma total deoxyribonucleic acid (DNA) levels in normotensive, healthy pregnant women and preeclamptic patients**. Middle point: median, whisker: 25-75 percentile.

The clinical characteristics of the studied pregnant women are shown in Table 1. The measured laboratory parameters were significantly different between the two groups, except for serum AST activities (Table 2). Quantifying the amount of Y chromosome-specific DNA, we were able to confirm that the levels of male fetal DNA were significantly elevated in pregnancies complicated by preeclampsia compared to the normotensive control subjects (median: 0.086 pg/μl vs. 0.001 pg/μl plasma; *P *< .001; Figure 1). Data regarding the quantity of total cell-free DNA (globin) also confirmed significant difference between the two groups (median: 32.460 pg/μl vs. 11.395 pg/μl plasma; *P *< .001; Figure 2). As BMI and gestational age at blood sampling, which are known to affect plasma DNA levels, differed significantly between the preeclamptic and the control group, adjusted values were calculated with analyses of covariance (ANCOVA). The differences in fetal and total cell-free DNA levels between the two study groups remained significant even after adjustment for maternal age, BMI and gestational age at blood draw in ANCOVA (Table 2).

**Table 1:** Clinical characteristics of normotensive, healthy pregnant women and preeclamptic patients

Variable	Controls (n = 70)	Preeclampsia (n = 67)	Statistical significance (p value)
Age (years)	30 (17-44)	29 (19-42)	NS

BMI at blood draw (kg/m^2^)	25.9 (19.0-42.0)	30.0 (20.6-38.3)	<0.001

Smokers	0 (0%)	3 (4.5%)	NS

Primiparas	45 (64.3%)	43 (64.2%)	NS

Systolic blood pressure (mmHg)	110 (80-138)	160 (135-220)	<0.001

Diastolic blood pressure (mmHg)	70 (55-86)	100 (90-131)	<0.001

Gestational age at blood draw (weeks)	35 (20-40)	38 (30-41)	<0.001

Gestational age at delivery (weeks)	39 (35-41)	38 (33-41)	<0.05

Fetal birth weight (grams)	3500 (2650-4400)	3200 (1400-4200)	<0.001

Fetal growth restriction	0 (0%)	11 (16.4%)	<0.001

Data are presented as median (range) for continuous variables and as number (percent) for categorical variables.

NS: not significant; BMI: body mass index

**Table 2:** Laboratory parameters of normotensive, healthy pregnant women and preeclamptic patients

Variable	Controls (n = 70)	Preeclampsia (n = 67)	Statistical significance (p value)
Serum BUN level (mmol/l)	2.7 (1.7-4.8)	3.4 (0.8-6.5)	<0.001

Serum creatinine level (μmol/l)	47 (34-79)	63 (36-95)	<0.001

Serum bilirubin level (μmol/l)	5.1 (1.8-15.2)	7.4 (2.1-20.9)	<0.001

Serum AST activity (U/l)	19 (10-38)	19 (10-148)	NS

Serum ALT activity (U/l)	12 (7-32)	15 (6-233)	<0.05

Serum LDH activity (U/l)	159 (93-211)	192 (113-403)	<0.001

Serum CRP level (mg/l)	3.6 (0.5-28.0)	6.7 (0.3-36.9)	<0.05

Plasma VWF:Ag level (%)	129.3 (47.8-297.1)	187.1 (43.3-423.0)	<0.001

Plasma fibronectin level (g/l)	0.33 (0.14-0.84)	0.58 (0.02-2.13)	<0.001

Plasma malondialdehyde level (nmol/ml)	14.74 (6.19-36.52)	18.62 (10.75-24.65)	<0.001

Plasma total DNA level (pg/μl)	11.395 (0.425-256.3)	32.460 (1.670-602.7)	<0.001

Log (total DNA level) (pg/μl) (adjusted mean ± SE)	1.145 ± 0.066	1.513 ± 0.086	<0.05

Plasma fetal DNA level (pg/μl)	0.001 (0.0-0.845)^†^	0.086 (0.002-3.200)^‡^	<0.001

Log (fetal DNA level) (pg/μl) (adjusted mean ± SE)	-2.705 ± 0.310^†^	-0.994 ± 0.326^‡^	<0.05

Data are presented as median (range) for continuous variables

NS: not significant; SE: standard error; BUN: blood urea nitrogen; AST: aspartate aminotransferase; ALT: alanine aminotransferase; LDH: lactate dehydrogenase; CRP: C-reactive protein; VWF:Ag: von Willebrand factor antigen; DNA: deoxyribonucleic acid

^† ^n = 25

^‡ ^n = 36

Correlation coefficients with p values between clinical characteristics and laboratory parameters of preeclamptic patients and plasma total DNA levels are presented in Table 3. Total cell-free DNA levels showed no significant correlations with the clinical characteristics of preeclamptic patients, including BMI. Furthermore, the quantity of total plasma free DNA did not correlate with most of the laboratory parameters including plasma cff DNA levels, except for serum aspartate aminotransferase and alanine aminotransferase activities (correlation coefficient: 0.31; *P *= 0.012 and 0.46; *P *< .001).

**Table 3:** Correlation coefficients with p values between clinical characteristics and laboratory parameters of preeclamptic patients and plasma total DNA levels

Variable	Correlation coefficient	Statistical significance (p value)
Age	-0.04	0.72

BMI at blood draw	0.18	0.23

Systolic blood pressure	-0.10	0.43

Diastolic blood pressure	-0.21	0.087

Gestational age at blood draw	0.10	0.40

Gestational age at delivery	0.02	0.87

Fetal birth weight	-0.01	0.96

Serum BUN level	-0.07	0.57

Serum creatinine level	0.03	0.83

Serum bilirubin level	0.06	0.62

Serum AST activity	**0.31**	**0.012**

Serum ALT activity	**0.46**	**<0.001**

Serum LDH activity	0.17	0.23

Serum CRP level	0.04	0.76

Plasma VWF:Ag level	-0.18	0.14

Plasma fibronectin level	0.01	0.96

Plasma malondialdehyde level	-0.11	0.36

Plasma fetal DNA level^‡^	0.14	0.41

BMI: body mass index; BUN: blood urea nitrogen; AST: aspartate aminotransferase; ALT: alanine aminotransferase; LDH: lactate dehydrogenase; CRP: C-reactive protein; VWF:Ag: von Willebrand factor antigen; DNA: deoxyribonucleic acid

^‡ ^n = 36

The sample size of the preeclamptic group allowed us to detect a correlation coefficient (rho) of 0.30 or higher, at a Type I error rate of 0.05, with a statistical power of at least 80%.

## Comment

Several papers described previously that fetal-maternal cell trafficking was significantly disturbed in pregnancies complicated by preeclampsia, with elevated numbers of fetal cells detected in the maternal circulation during these pregnancies [11-13]. Lo et al. [14] showed that the fetal circulating DNA level was elevated in a similar manner during pregnancies complicated by preeclampsia [5]. We were able to confirm the latter observation in this study by showing that the levels of circulating total and fetal DNA were significantly elevated in women with preeclampsia compared to the control group. Our study also confirmed that the quantification of fetal and total circulating DNA by real-time PCR was both reliable and reproducible. In addition to previous findings, with collected data regarding clinical characteristics and measuring different laboratory parameters specific to renal and liver function, inflammation, endothelial activation/damage and oxidative stress, we were able to analyze the correlation between the quantity of cell-free DNA and these parameters first in the literature. Although the exact mechanism leading to the release of free extracellular DNA into the circulation is not yet clear [15], currently the most favored explanation for the release of DNA fragments is through apoptosis or some other form of cell death [16-18]. Some other studies, however, have indicated that a normal physiological process may be involved in the release of free circulating DNA [15]. In contrast to Zhong et al. [8], we did not find a correlation between cf DNA and cff DNA levels, which indicates that the amount of circulating total and fetal DNA does not increase similarly in preeclampsia. Indeed, the median plasma level of total DNA was 2.85 times higher in our preeclamptic group, while that of fetal DNA elevated 86-fold in preeclampsia. The latter seems to reflect extensive placental injury in the disease. The clinical characteristics of our preeclamptic patients, including body mass index, did not affect plasma total DNA concentrations, which is at variance with previous observations. Our data did not confirm any correlation between the markers of endothelial cell activation/damage and the level of total cell-free DNA, which suggests that the elevation of the DNA amount is caused by another mechanism and the previously suggested endothelial dysfunction is not the primary cause that leads to the increased plasma DNA level observed in preeclampsia. Inflammatory cytokines and reactive oxygen species might damage cellular membranes and DNA in preeclampsia. Nevertheless, total cell-free DNA levels were not related to markers of inflammation and oxidative stress in our preeclamptic group. Instead, only liver enzyme activities showed significant correlations with plasma total DNA levels, which suggests that hepatocellular necrosis might account - at least in part - for increased circulating total DNA levels in preeclampsia. Interestingly, plasma total DNA levels were found previously to be significantly higher in HELLP syndrome than in preeclampsia without HELLP syndrome [19]. HELLP syndrome is characterized by extensive tissue damage (hepatocellular necrosis and hemolysis), which supports the hypothesis that cellular necrosis might be responsible for increased cell-free DNA levels in the maternal circulation, at least in preeclampsia. However, it is also possible that another unrelated mechanism, such as release from activated granulocytes and other white blood cells, accounts primarily for the increase in circulating DNA levels associated with preeclampsia. The lack of correlation between increased circulating total DNA levels and several measured laboratory markers in preeclampsia could not completely exclude the relationship of cell-free DNA with the corresponding pathological processes. The release rate, clearance rate and half-lives of the investigated markers may be different from those of cell-free DNA, which might also explain the lack of correlation. More detailed studies will be necessary to elucidate the underlying mechanisms and biochemical background of this medically relevant disorder. Our results provide some new data for further investigations.

## Competing interests

The authors declare that they have no competing interests.

## Authors' contributions

LL made substantial contributions to conception and design of study and carried out the molecular genetic studies. RJJr made the manuscript revising. NB carried out the molecular genetic studies. BK carried out the immunoassays. MVcarried out the sample collection. CL made the analysis and interpretation of data. MM participated in its design and coordination. PZ carried out the immunoassays. MA has participated in the design of the study and performed the statistical analysis. All authors read and approved the final manuscript.

## Pre-publication history

The pre-publication history for this paper can be accessed here:

http://www.biomedcentral.com/1471-2350/10/120/prepub
